# Expanding on roles of pleckstrin homology-like domain family A member 1 protein

**DOI:** 10.1007/s00441-024-03942-2

**Published:** 2024-12-04

**Authors:** Małgorzata Durbas

**Affiliations:** Laboratory of Molecular Genetics and Virology, Faculty of Biochemistry, Biophysics and Biotechnology, Jagiellonian University, Krakow, Poland

**Keywords:** PHLDA1, Cancer, Apoptosis, Autophagy, Pyroptosis

## Abstract

Pleckstrin homology-like domain, family A, member 1 (PHLDA1), one of the three members of PHLDA (1–3) family, has been reported to be expressed in mammalian cells and tissues and play diverse roles in various biological processes such as apoptosis, pyroptosis, and differentiation. Nevertheless, new roles and mechanisms of PHLDA1 action have come to light, with some needing further clarification. The major aim of the publication is to review proapoptotic or antiapoptotic roles of PHLDA1 in cancer, including ample evidence on *PHLDA1* role as a tumor suppressor gene or oncogene and its influence on tumor progression. The role of PHLDA1 as a prognostic marker of cancer emerges, as well as its role in drug response and resistance. PHLDA1 involvement in autophagy, endoplasmic reticulum stress, pyroptosis, or differentiation is also scrutinized. It is also important to note that the association of PHLDA1 with miRNA regulation is described. Additionally, the emerging functions of PHLDA1 are indicated, specifically in inflammation and ischemia/reperfusion injury.

## A synopsis of PHLDA1

**Fig. 1:**
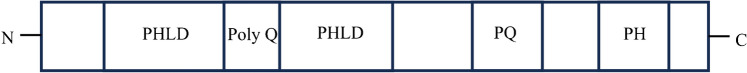
PHLDA1 structure. N—amino-terminal region, PHLD—pleckstrin homology-like domain; Poly Q—glutamine-rich sequence; PQ—proline-glutamine-rich tract; PH—proline-histidine-rich tract; C—carboxy-terminal region

The human *PHLDA1* gene encodes for a 260 amino acid protein. The murine *PHLDA1* gene (*TDAG51*) encodes for a 261 amino acid protein, and its product was first identified as a potential transcription factor required for T cell receptor (TCR) activation-induced apoptosis in mouse T-cell hybridomas (Park et al. 1996). More light on the function of PHLDA1 can be shed through analysis of its structure, as presented in Fig. 1. Thus, the protein in its N-terminal part contains a pleckstrin homology-like domain (PHLD) with the property to bind phosphatidylinositol lipids and proteins (Lemmon and Ferguson 2000). It is common for proteins with the PHL domain to interact with membrane components, which affects signal transduction (mediating protein–protein interactions) and cytoskeletal organization (actin assembly, cell polarization) (Lemmon et al. 2002; Scheffzek and Welti 2012). The PHL domain is spanned by a glutamine-rich sequence (Poly Q). PHLDA1 also contains proline-glutamine (PQ) and proline-histidine-rich (PH) tracts in its C-terminal region (Fig. 1). The expression of PQ/PH-rich proteins has been involved in transcriptional regulation and apoptosis in neuronal cells (Gomes et al. 1999).

PHLDA1 is expressed and localized differently in mammalian tissues. High expression levels of *PHLDA1* mRNA were detected in the lung and pancreas, and moderate expression in the brain, heart, placenta, liver, and kidney, as shown by northern blot analysis of human tissue samples (Hossain et al. 2003; Ohyama et al. 2006). High levels of PHLDA1 protein were reported in mouse lungs, and they were only moderate in liver, thymus, and adipocytes (Basseri et al. 2013). In cancer, the lowest level of PHLDA1 protein was found in leukemia-derived cell lines and the highest in neuroblastoma, as shown by analysis of Cancer Cell Line Encyclopedia proteomics dataset of 378 samples from 24 different cancer types (Bugara et al. 2024). PHLDA1 subcellular localization may be associated with various functions in a tissue- and cell-dependent manner (Neef et al. 2002; Nagai et al. 2007; Yousof et al. 2023). In metastatic melanoma, strong cytoplasmic staining of PHLDA1 results in apoptosis and growth dysregulation (Neef et al. 2002). Localization of PHLDA1 in the cytoplasm and nucleus of T-cells inhibits protein synthesis (Hinz et al. 2001). PHLDA1 is regulated by numerous stimuli, such as growth factors, endoplasmic reticulum (ER) stress- and differentiation-inducing agents (Table 1). Several common drugs such as acetaminophen (paracetamol), cyclosporine, or cisplatin could also potentially modulate PHLDA1 expression as indicated by the PHLDA1-drug interaction network generated using Comparative Toxicogenomics Database (Baldavira et al. 2021). Also, a study by Duan et al. using Gene Set Cancer Analysis Lite database revealed both positive and negative regulation of PHLDA1 by chemotherapeutic or targeted drugs (Duan et al. 2022).

**Table 1:** Positive and negative regulation of PHLDA1

Positive regulation of PHLDA1	References
ER-stress inducing agents (homocysteine, farnesol, thapsigargin)	Hossain et al. 2003; Joung et al. 2007; Carlisle et al. 2012; Xu et al. 2021
Growth factors (EGF, FGF, PDGFB, IGF-1^1^)	Gomes et al. 1999; Toyoshima et al. 2004; Wu et al. 2010; Fearon et al. 2018
Phorbol esters	Wang et al. 1998; Hinz et al. 2001; Oberg et al. 2004
Ionomycin	Hinz et al. 2001
Heat shock factor 1	Hayashida et al. 2006
Drugs (acetaminophen, cyclosporine, selumetinib, trametinib)	Baldavira et al. 2021; Duan et al. 2022
Negative regulation of PHLDA1	References
Aurora A	Johnson et al. 2011; Durbas et al. 2016
AKT	Magi et al. 2018
Drugs (cisplatin, docetaxel, gemcitabine)	Baldavira et al. 2021; Duan et al. 2022

^1^*EGFR* epidermal growth factor receptor, *FGFR* fibroblast growth factor receptor, *PDGFB* platelet-derived growth factor subunit B, *IGF-1* insulin-like growth factor

## PHLDA1 in cancer

**Fig. 2:**
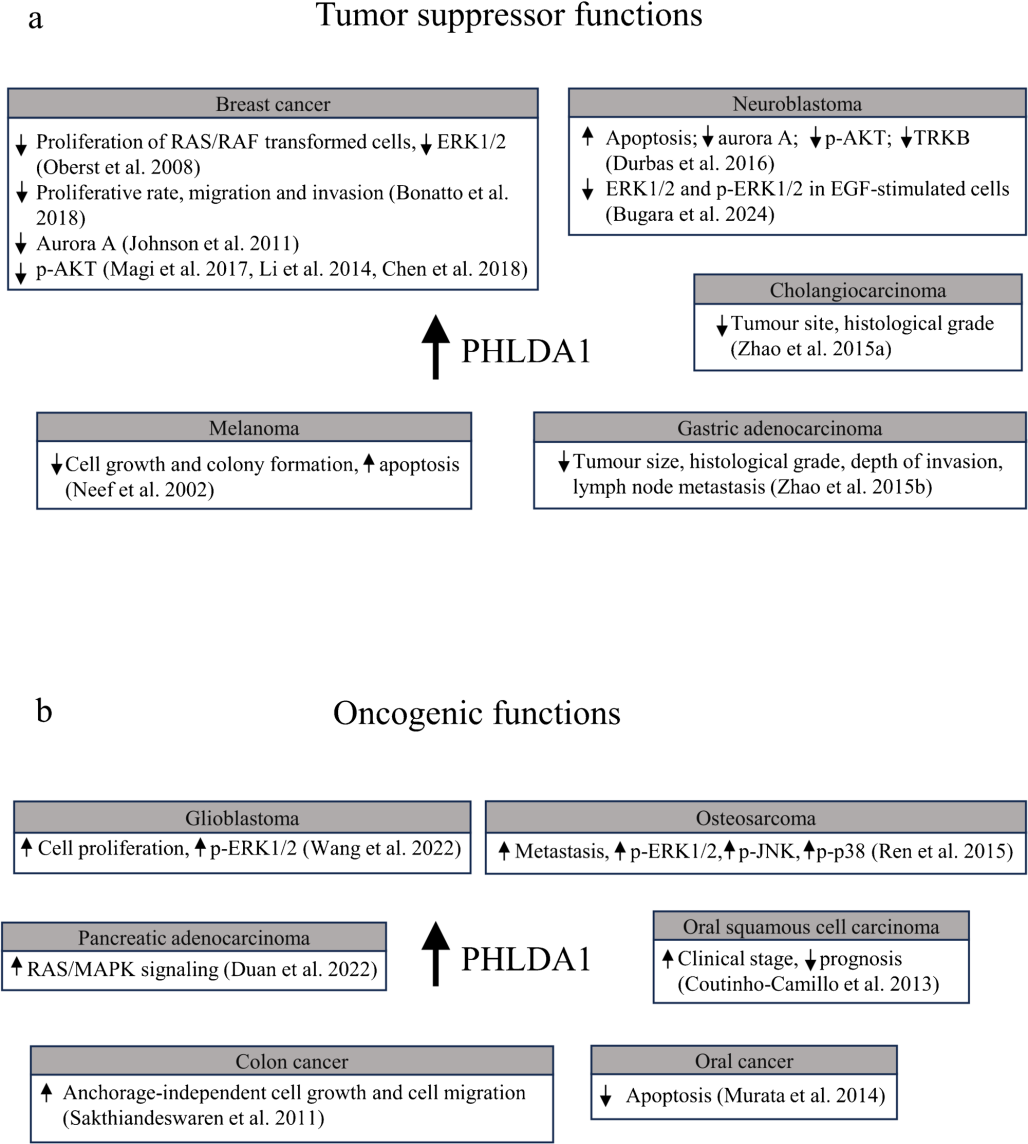
Tumor suppressor and oncogenic functions of PHLDA1 in cancer models. **a** Tumor suppressor functions of PHLDA1 in breast cancer, neuroblastoma, cholangiocarcinoma, gastric adenocarcinoma, and melanoma. **b** Oncogenic functions of PHLDA1 in glioblastoma, osteosarcoma, oral squamous cell carcinoma, oral cancer, colon cancer, and pancreatic adenocarcinoma. p-phospho; ERK1/2 – extracellular signal-regulated kinase 1/2; TRKB – tropomyosin receptor kinase B; EGF – epidermal growth factor; JNK – c-Jun N-terminal kinase; MAPKs – mitogen-activated protein kinases

The PHLDA1 protein plays essential roles in cancer, depending on the cellular type and context. It regulates oncogenic pathways, growth factor signalling, and affects apoptosis. The PHLDA1 can act as a tumor suppressor, but oncogenic roles were also reported in some cancers, as presented in Fig. 2.

### Tumor suppressor functions

The literature broadly describes the tumor suppressor functions of PHLDA1 (Nagai 2016; Yousof et al. 2023). They mainly rely on the induction of apoptosis in cancer, the inhibition of mitogenic pathways such as PI3K/AKT and RAS/RAF/MEK/MAPK signaling, and the inhibition of invasion and metastasis. The tumor suppressor functions of PHLDA1 were revised in different types of cancer in Fig. 2a.

The dysregulation of the RAS/RAF/MEK/MAPK pathway is a common characteristic of tumor cells, and it promotes tumorigenesis by affecting cell growth, division, migration, or differentiation (Bahar et al. 2023). *RAS* is the most frequently mutated oncogene in cancer, and RAF is a mediator of MAPK signaling that leads to the activation of extracellular signal-regulated kinase (ERK). In a report concerning breast cancer, the upregulation of PHLDA1 in HME16C mammary epithelial cells inhibited ERK-mediated transformation. In contrast, the knockdown of PHLDA1 resulted in an increased ERK pathway activation and enhanced cellular proliferation of RAS/RAF transformed cells under anchorage-independent conditions (Oberst et al. 2008). Another report by Bonatto showed that PHLDA1 downregulation promoted colony formation, migration, and invasion of MCF10A breast epithelial cells (Bonatto et al. 2018). In another study, PHLDA1 upregulation inhibited cell proliferation and motility, as shown by reducing chemotaxis in MDA-MB-231 breast cancer cells (Johnson et al. 2011). Noteworthy, PHLDA1 was identified as an important negative regulator of aurora A kinase in breast cancer as it contributes to the degradation of this oncoprotein. The group also reported that PHLDA1 is a substrate of aurora A in MDA-MB-231 cells, as shown in in vitro kinase assays, and aurora A kinase phosphorylates PHLDA1 at Ser98, leading to its degradation. Thus, PHLDA1 degradation might be one of the mechanisms of enhancing cell proliferation by which aurora A promotes breast malignancy.

The dysregulation of the ErbB receptor pathway is associated with tumor progression (Hynes and MacDonald 2009). Downstream targets of ErbB receptors are ERK and AKT, which subsequently affect the transcriptional regulation (Roy et al. 2010; Wee and Wang 2017). Magi et al. found that one of the ErbB3 ligands, heregulin, induces *PHLDA1* mRNA in breast adenocarcinoma MCF-7 cells (Magi et al. 2018). This induction involves both RAS/ERK and PI3K/AKT pathways because PHLDA1 overexpression suppressed the phosphorylation of AKT and ERK1/2 kinases. Moreover, PHLDA1 protein binds directly to ErbB3 as assessed by co-immunoprecipitation and immunobloting and negatively regulates ErbB receptor signaling by inhibiting ErbB3 and ErbB2 receptor complex formation and its subsequent transactivation. In another study by Li et al. PHLDA1 was shown to be downregulated after ErbB2 signaling inhibition by lapatinib (inhibitor of ErbB1/EGFR and ErbB2), CL387,785, and erlotinib (inhibitors of ErbB1) in SKBR3 cells (Li et al. 2014). Additionally, a significant downregulation of PHLDA1 in primary human breast cancer was found by immunohistochemical staining and PHLDA1 was significantly less expressed in ErbB2-negative tumors as compared with ErbB2-positive tumors, where PHLDA1 was expressed abundantly. In the same work, the authors showed that PHLDA1 overexpression in SKBR3 cells blocked AKT signaling by inhibiting AKT phosphorylation, inhibiting cell growth, colony-forming ability, and cell motility. Additionally, the overexpression of PHLDA1 enhanced cells sensitivity to lapatinib, and caused 50% inhibition of colony formation in breast cancer. Similar negative regulation between PHLDA1 and AKT was also reported in IMR-32 neuroblastoma cells (Durbas et al. 2016). Hence, cells with downregulated PHLDA1 showed an increase in AKT phosphorylation. Another observation was that *PHLDA1*-silenced IMR-32 cells were less susceptible to apoptosis, as demonstrated by decreased levels of cleaved forms of caspase 3 and its substrate poly (ADP-ribose) polymerase, PARP, and decreased activities of caspase 3 and 7. The downregulation of PHLDA1 also led to a significant increase in the expression of tropomyosin receptor kinase B (TRKB) and aurora A kinase, which are markers of poor prognosis in neuroblastoma (Brodeur et al. 2009; Durbas et al. 2016). The group also reported that IMR-32 cells with downregulation of PHLDA1 showed enhanced cellular ATP levels and increased mitochondrial membrane potential (Durbas et al. 2016). Moreover, Bugara et al. performed quantitative proteomic analysis of *PHLDA1*-silenced IMR-32 neuroblastoma cells, which revealed the most pronounced changes in mitochondrial proteome as compared to control cells, established by gene ontology annotation analysis using the DAVID 6.8 platform (Wei Huang et al. 2009; Bugara et al. 2024). Also, in this neuroblastoma model, the downregulation of PHLDA1 caused the increased levels of ERK1/2 in epidermal growth factor (EGF)-treated cells and non-treated cells as well as increased phosphorylation of ERK1/2 in EGF-stimulated cells.

Tumor suppressor functions of PHLDA1 are also evident in cholangiocarcinoma and gastric adenocarcinoma (Zhao et al. 2015a, b). In cholangiocarcinoma, a negative correlation was found between the PHLDA1 expression and such clinicopathological features as tumor site and histological grade (Zhao et al. 2015a). Also, in gastric adenocarcinoma, the expression of PHLDA1 protein was correlated with less malignant phenotype, e.g., decreased invasion and metastasis, decreased tumor size, and lower histological grade (Zhao et al. 2015b).

In conclusion, the oncosuppresor functions of PHLDA1 are broadly documented in a variety of tumors such as breast cancer, neuroblastoma, cholangiocarcinoma, gastric adenocarcinoma, or melanoma. This shows the potential of PHLDA1 upregulation for promoting tumors cell death, supressing key oncogenic pathways, and developing a malignant phenotype.

### Oncogenic role of PHLDA1

It should be stressed that the oncogenic roles of PHLDA1 were also reported by several groups, and they were summarized in different types of cancer in Fig. 2b. Wang et al. reported that PHLDA1 acted as an oncogene as the overexpression of the protein increased cell proliferation and colony-forming ability in HEB and LN229 glioblastoma cells (Wang et al. 2022a). The group also reported that PHLDA1 activates the RAS/RAF/MEK/ERK signalling pathway, as the downregulation of PHLDA1 in U87 and U251 cells led to lower phosphorylation levels of ERK1/2 and its downstream target, ribosomal protein S6 kinase A1 (RSK-1), as assessed by immunoblotting. Similarly, the experiments on PHLDA1 knockout mice demonstrated that the phosphorylation status of ERK1/2 and RSK-1 is decreased. Most importantly, the group examined the presence of PHLDA1 and RAS interaction by immunoprecipitation assay. Additionally, PHLDA1 promoted RAS activity as assessed by kinase assay. Ren et al. showed that elevated *PHLDA1* expression correlated with high metastatic potential in osteosarcoma cells, as indicated by DNA microarrays and confirmed by RNA-seq (Ren et al. 2015). However, *PHLDA1* silencing did not affect the viability or proliferation rate of MG63.3 osteosarcoma cells. Mechanistically, the authors showed that *PHLDA1* silencing resulted in the downregulation of the activities of MAPKs, *i.e.*, ERK1/2, c-Jun N-terminal kinases (JNK), and p38 mitogen-activated protein kinase in MG63.3 osteosarcoma cells, as shown by kinase array assay. Additionally, in oral squamous cell carcinoma, tissue microarray analysis followed by immunohistochemistry revealed that the expression of PHLDA1 corresponded to a higher clinical stage and, therefore, a worse prognosis for patients (Coutinho-Camillo et al. 2013). It was also reported that the knockdown of PHLDA1 in Ca9-22 oral cancer cells increased apoptosis, as evidenced by increased levels of active caspase 3 and its substrate, cytokeratin 18 (Murata et al. 2014). Finally, a study by Sakthianandeswaren et al. revealed that silencing of *PHLDA1* in HCT116 and SW480 colon cancer cells inhibited anchorage-independent cell growth and cell migration as assessed by colony formation assay and chamber migration assay, respectively (Sakthianandeswaren et al. 2011). The reduction of tumor growth was also observed in mice xenograft models after injecting HCT116 cells with downregulated expression of *PHLDA1.* Finally, the group of Duan et al. presented PHLDA1 as an activator of RAS/MAPK signaling in pancreatic adenocarcinoma as a result of examining the GSCALite database (Duan et al. 2022). Overall, PHLDA1 roles, described as oncogenic, mainly manifest by inducing cell proliferation and migration and activating RAS/MAPK signaling.

The role of PHLDA1 in cancer is the best characterized as dichotomous, demonstrating either oncosuppressor or oncogenic features depending on the type of tumor.

## PHLDA1 as a prognostic marker

PHLDA1 was evaluated as a prognostic marker in several types of cancer. PHLDA1 is a potential prognostic marker of patient survival in breast cancer (Nagai et al. 2007). Downregulation of PHLDA1 was associated with poor prognosis, more advanced clinical stage, and decreased overall survival (OS), as shown by the analysis of Kaplan–Meier curves in breast cancer patients. Additionally, decreased expression of PHLDA1 in gastric adenocarcinoma correlates with decreased overall survival and increased tumor size, grade and metastasis (Zhao et al. 2015b). However, the multivariate analysis performed by the group did not indicate PHLDA1 as an independent prognostic factor. The prognostic significance of PHLDA was also found in non-small lung cancer, where low *PHLDA1* mRNA expression was correlated with a worse OS in the Kaplan–Meier analysis (Baldavira et al. 2021). In contrast, the group showed that mesothelioma patients with high *PHLDA1* mRNA expression had poor OS (Baldavira et al. 2021). Moreover, expression of PHLDA1 as analyzed by immunohistochemistry in tissue microarray was associated with an advanced clinical stage and histological grade of oral squamous cell carcinomas and was postulated to be an independent prognostic factor by multivariate analysis (Coutinho-Camillo et al. 2013). Another study showed the high expression of PHLDA1/3 as assessed by immunohistochemical staining in pancreatic adenocarcinoma tissues and using the Human Protein Atlas database. The high expression of PHLDA1 was associated with decreased overall survival, relapse-free survival, and a poor prognosis in patients with pancreatic adenocarcinoma (Duan et al. 2022). Interestingly, the OS was even more shortened in patients with a high frequency of *PHLDA1* mutations as determined using the cBioPortal database (Gao et al. 2013; Duan et al. 2022). Another finding showed that PHLDA1 protein levels exhibited a prognostic value in glioblastoma (Wang et al. 2022a). Thus, the OS and disease-free survival were markedly shortened in tumors with high PHLDA1 protein levels compared to low PHLDA1 levels. High PHLDA1 levels were also associated with a greater tendency of disease recurrence.

The results above indicate that the PHLDA1 protein is a new prognostic marker. The prognostic value of PHLDA1 in cancer was summarized in Table 2, showing that the expression level of PHLDA1 is associated with contrasting effects on disease prognosis and patient overall survival depending on the type of tumor. These results emphasize the conclusion that PHLDA1 might be an attractive therapeutic target in breast cancer, pancreatic, gastric adenocarcinoma, and glioma, provided that its expression profile is determined.

**Table 2:** The prognostic value of PHLDA1 in various types of cancer

Expression of PHLDA1	Type of cancer	Prognostic values	References
↓PHLDA1	Breast cancer	↓Prognosis ↓Overall survival	Nagai et al. 2007
	Gastric adenocarcinoma	↓Overall survival	Zhao et al. 2015b
	Non-small cell lung cancer	↓Overall survival	Baldavira et al. 2021
↑PHLDA1	Mesothelioma	↓Overall survival	Baldavira et al. 2021
	Oral squamous cell carcinoma	↓Prognosis ↓Disease-free survival	Coutinho-Camillo et al. 2013
	Pancreatic adenocarcinoma	↓Prognosis ↓Overall survival	Duan et al. 2022
	Glioblastoma	↓Overall survival ↓Disease-free survival	Wang et al. 2022a

## PHLDA1 participates in drug response and resistance

The effect of targeted therapies might be limited by the development of tumor resistance to the treatment (Ayati et al. 2020). Some groups reported that PHLDA1 not only participates in the drug response but could also be a modulator of the process of developing resistance to drugs.

Epidermal growth factor receptor (EGFR/ErbB1) and fibroblast growth factor receptor (FGFR) signalling pathways are frequently overactivated in tumors, and their inhibition by their specific receptor tyrosine kinase inhibitors is associated with evolving drug resistance (Carter et al. 2015; Ayati et al. 2020; Chhouri et al. 2023). Using monoclonal antibodies is also a clinically important pharmacological approach in anti-EGFR and anti-FGFR therapies (Ayati et al. 2020; Du et al. 2023). The role of PHLDA1 is especially evident in the case of treatment with drugs being inhibitors of EGFR and FGFR and monoclonal antibodies targeting these receptors. The group of Fearon et al. reported that the *PHLDA1* gene was significantly downregulated by PD173074 and AZD4547 (both inhibitors of FGFR) in MFE-296 endometrial adenocarcinoma cells as identified by microarrays (Fearon et al. 2018). Moreover, they showed that the knockdown of PHLDA1 rendered MFE-296 cells more resistant to AZD4547 treatment, whereas the re-expression of PHLDA1 made cells more sensitive. The group also showed that treating SKBR3 and HCC1954 breast cancer cells with lapatinib (inhibitor of EGFR/ErbB1 and ErbB2) led to a decrease in PHLDA1 as assessed by immunoblotting. Also, in this case, reversing the situation by recovering PHLDA1 expression contributed to sensitizing the cells to lapatinib. Therefore, it was concluded that the observed downregulation of PHLDA1 is a universal phenomenon associated with the action of inhibitors of receptor tyrosine kinases and, in consequence, evokes resistance to the treatment. A similar effect was also observed in breast cancer for treatment with trastuzumab, monoclonal antibodies targeting EGFR (Fearon et al. 2018). The MCF7/HER2-18 cells with PHLDA1 downregulation were shown to be more resistant to trastuzumab. The findings above were further expanded by Clayton et al., who used lapatinib combined with 4SC-202, the inhibitor of histone deacetylase 1/2/3 (HDAC1/2/3) (Clayton et al. 2023). The inhibition of HDAC by 4SC-202 restored the PHLDA1 expression in lapatinib-resistant SKBR3 and HCC1954 breast cancer cells. Therefore, this dual treatment increased the sensitivity of these cell lines to lapatinib. These studies emphasize the potential of PHLDA1 restoration/re-expression for the augmentation of sensitivity to EGFR and FGFR-targeted drugs. Another contrasting function of PHLDA1 emerged in HCT116, HT29, and SW48 colorectal cancer cells, where PHLDA1 upregulation was associated with resistance to cetuximab, a monoclonal antibody targeting EGFR (Park et al. 2019). The study showed that the knockdown of PHLDA1 increased the sensitivity of SW48 cells to cetuximab. A similar response was observed for treatment with erlotinib (an inhibitor of EGFR). Thus, the knockdown of PHLDA1 rendered SW48 cells more sensitive to erlotinib. Another finding by Bugara et al. showed that the downregulation of PHLDA1 in IMR-32 neuroblastoma cells does not affect the response to treatment with lapatinib and another inhibitor of EGFR, gefitinib (Bugara et al. 2024). Overall, the presented findings demonstrate that PHLDA1 is involved in response to EGFR and FGFR-targeted drugs. However, its influence on drug sensitivity or resistance is drug- and tumor-dependent.

The significance of PHLDA1 was also observed in ch14.18/CHO and 14G2a monoclonal antibodies response, which are GD2 ganglioside-targeting drugs in neuroblastoma. Anti-GD2 ganglioside 14G2a monoclonal antibody (mAb) induced PHLDA1 in IMR-32 neuroblastoma cells, and this event accompanied 14G2a antibody-induced cell death (Horwacik et al. 2013). The group also reported that silencing of *PHLDA1* affected the sensitivity of IMR-32 clones to treatment with 14G2a monoclonal antibody (Durbas et al. 2016). The silencing of *PHLDA1* caused the increased resistance to 14G2a mAb in the *PHLDA1*-silenced clone of IMR-32 cells compared to the respective controls. However, it was reported that susceptibility of *PHLDA1*-silenced IMR-32 clones to small molecule aurora A kinase inhibitor (MK-5108), PI3K inhibitor (LY294002), and dual PI3K/mTOR inhibitor (BEZ-235) remained unchanged as compared to control cells.

The expression of PHLDA1 in drug response and its regulation was also scrutinized based on the bioinformatic analyses. Regarding drug response, Baldavira et al. used the Comparative Toxicogenomics Database to analyse the potential intercorrelation between the *PHLDA1* gene and various chemical drugs (Baldavira et al. 2021). PHLDA1-drug interaction network showed that several commonly used drugs could modulate *PHLDA1* mRNA and protein. According to the analysis, cisplatin (chemotherapeutic drug) was found to negatively regulate PHLDA1, whereas acetaminophen (paracetamol) or cyclosporine (immunosuppressive agent) positively regulated the level of PHLDA1 (Table 1). Another bioinformatical analysis was performed on pancreatic adenocarcinoma by Duan et al., who presented the correlation between PHLDA1 expression and sensitivity to chemotherapeutic and targeted drugs used to treat pancreatic adenocarcinoma (Duan et al. 2022). A negative correlation was found between PHLDA1 and anti-cancer drugs such as docetaxel and gemcitabine, as researched in the GSCALite database. The analysis also revealed the positive regulation between PHLDA1/2 and MEK1/2 inhibitors such as selumetinib, trametinib, and PD318088 (Table 1). However, additional experimental and clinical evidence is needed to evaluate if the *PHLDA1* gene may be a reliable prognostic indicator of drug response, as suggested by these in silico results.

## Involvement of PHLDA1 in apoptosis, autophagy, and pyroptosis

The process of programmed cell death, or apoptosis, is critical for normal physiological processes. It relies on many signalling pathways, including caspases, leading to rapid cell demise (Pistritto et al. 2016). It is manifested by cellular shrinkage, nuclear chromatin condensation, blebbing, and apoptotic bodies. Abnormal apoptosis occurs in many human conditions, including many types of cancer, neurodegenerative diseases, ischemic damage, and autoimmune disorders (Kar and Sivamani 2015). The role of PHLDA1 in the process of apoptosis is still controversial. PHLDA1 was shown to act as a proapoptotic or antiapoptotic agent, depending on the cell type, disease state, and cellular context. The evidence of different PHLDA1 functions in apoptosis is presented in stress-induced cells and disease. The proapoptotic function of PHLDA1 was reported by Hayashida et al., who showed that apoptosis was increased when heat shock was applied to the testicles of wild-type mice as compared to PHLDA1-null mice (Hayashida et al. 2006). Therefore, PHLDA1 played an important role in the induction of apoptosis in mouse male germ cells in response to heat shock. In contrast, Park et al. showed the antiapoptotic function of the protein, as PHLDA1 deficiency (PHLDA1^−^/^−^) promoted oxidative stress-induced apoptotic cell death in mouse embryonic fibroblasts (Park et al. 2013). Toyoshima also identified an antiapoptotic role of PHLDA1 in NWTB3 mouse fibroblast cells, where silencing of *PHLDA1* enhanced serum starvation-induced apoptosis (Toyoshima et al. 2004). The proapoptotic or antiapoptotic roles of PHLDA1 were also widely reported in diseases and linked to endoplasmic reticulum (ER) stress. ER stress results from an accumulation of improperly folded polypeptides in the ER lumen (Fu and Doroudgar 2022). The accumulation of misfolded proteins causes the translocation of the chaperones to proteins assembled in the ER and the activation of several signalling pathways responsible for protein degradation and cellular apoptosis. The proapoptotic role of PHLDA1 was reported in chronic kidney disease and associated with ER stress (Carlisle et al. 2021). It was revealed that PHLDA1 induced apoptosis in the mouse kidney through a C/EBP-homologous protein (CHOP), involved in ER stress. A similar role of PHLDA1 was stated in ischemia–reperfusion injury (Liu et al. 2023). It was shown that PHLDA1 knockdown inhibited apoptosis in neurons affected by oxygen–glucose deprivation/reoxygenation, and decreased levels of CHOP. On the contrary, an antiapoptotic role of PHLDA1 was demonstrated by Xu et al., who showed that PHLDA1 reduced the susceptibility to apoptosis via the ER stress response pathway in human ovarian cancer cells (Xu et al. 2021). Xu et al. showed that *PHLDA1* silencing enhanced apoptosis in 2008 and SKOV3 cells induced by the ER stress inducer, thapsigargin. Additionally, the same cell lines models exposed to oxidative stress-inducing H_2_O_2_ were characterized by increased expression of ER stress-associated proteins such as protein-disulfide isomerase (PDI), binding immunoglobulin protein (BIP) and inositol-requiring protein 1 (IRE1α). Therefore, PHLDA1 functions are inherent in apoptosis and apoptosis-related stresses in diseases.

A counterpart to apoptosis is autophagy, a catabolic process that allows the recycling of degraded cellular components to maintain nutrient and energy homeostasis (Yang et al. 2011). The autophagic response has been described in various pathophysiological situations, including cancer, cardiovascular disease, and infectious diseases. There is a strong correlation between defective autophagy and the formation of cancer, and studies have shown that several proteins and pathways related to autophagy are deregulated during cancer development (Aredia et al. 2012). However, its role in diseases is not fully understood as it can act in different conditions to work towards cell survival or cell death (Galluzzi et al. 2015). The findings about the role of PHLDA1 in the autophagy process are limited in the tumors. However, they are usually described in relation to apoptosis. Table 3 presents the effects of PHLDA1 downregulation on regulating apoptosis and autophagy in cancer cell lines. As a matter of fact, *PHLDA1* was found to be up-regulated in both autophagy and apoptosis induced by rapamycin in T-47D breast carcinoma (Moad et al. 2013). The silencing of this gene resulted in a reduction of these processes, as shown by the decrease of apoptotic markers such as cleaved caspase 3, BAX, and BAK, and a key autophagy marker, microtubule-associated protein 1 light chain 3 alpha/beta (LC3A/B-II). Durbas et al. also reported that the downregulation of PHLDA1 in IMR-32 neuroblastoma cells rendered these cells more resistant to apoptosis, as shown by decreased levels of cleaved caspase 3 and its substrate PARP and decreased activity of caspase 3/7 in *PHLDA1*-silenced cells as compared to control cells (Durbas et al. 2016). However, in IMR-32 neuroblastoma cells with downregulated PHLDA1, the expression of autophagy marker LC3A/B-II was increased (Durbas et al. 2016). Our group also showed that in another neuroblastoma cell line, CHP-134, the PHLDA1 protein negatively regulates apoptosis and positively regulates autophagy, showing some differences even between neuroblastoma cell lines (Durbas et al. 2018). In contrast, other studies by the group of Xu et al. showed that shPHLDA1-mediated knockdown did not alter the expression of autophagy-related proteins (LC3, beclin-1, P62) in ovarian 2008 cells exposed to H_2_O_2_, even though it stimulated apoptosis (Xu et al. 2021). The role of PHLDA1 in apoptosis and autophagy is interconnected but not uniform, depending on the type of tumor and, in some instances, even the cell line.

**Table 3:** Downregulation of PHLDA1 has an impact on the regulation of various cell death types in cancer cell lines and tissues

	Cell lines/tissues	Type of cell death	References
Cancer cell lines	T-47D cell line (breast cancer)	↓apoptosis, ↓autophagy	Moad et al. 2013
	IMR-32 cell line (neuroblastoma)	↓apoptosis, ↑autophagy	Durbas et al. 2016
	CHP-134 cell line (neuroblastoma)	↑apoptosis, ↓autophagy	Durbas et al. 2018
	2008 cell line (ovarian cancer)	↑apoptosis, -autophagy	Xu et al. 2021
Tissues	Intestinal tissues of neonatal mice	↓pyroptosis	Yang et al. 2023
	Lung tissues of mice	↓pyroptosis	Meng et al. 2023
	HT-22 mice neuronal cells	↓pyroptosis	Wang et al. 2024
	Neuronal cells of neonatal rats	↓pyroptosis, ↓apoptosis	Shu and Du 2022

Pyroptosis is an inflammatory caspase-dependent form of programmed necrosis that occurs in response to microbial infection (Tait et al. 2014). Morphologically, pyroptotic cells display cell swelling and rapid plasma membrane lysis. This form of cell death is driven by the inflammatory caspases: caspases 1, 4, 5, and 11 (Tait et al. 2014). In the inflammasome pathway, sensor proteins, including nod-like receptors (NLRs), detect pathogens and inflammatory agents (Man et al. 2017). After activation, NLR family pyrin domain-containing 3 protein (NLRP3) recruits the apoptosis-associated speck-like proteins containing a CARD (ASC) protein. This complex dimerizes and activates caspase 1. Once caspases 1, 11, 4, or 5 have been activated, they trigger pyroptosis by cleaving gasdermin D (Man et al. 2017). Several groups documented the role of PHLDA1 in pyroptosis. It was reported that the knockdown of PHLDA1 led to the inhibition of pyroptosis in mice’s intestinal and lung tissues as well as neuronal cells (Meng et al. 2023; Yang et al. 2023; Wang et al. 2024). The inhibition of pyroptosis in these cells and tissues was clearly manifested by decreased levels of pyroptosis-associated proteins such as NLRP3, ASC and the cleavage of caspase 1 and gasdermin D. These events were accompanied by decreased intestinal, lung, and neuronal inflammation, as shown by reduced levels of IL-1β and IL-18. In these reports, PHLDA1 emerges as a positive regulator of pyroptosis. The role of PHLDA1 downregulation on the regulation of pyroptosis in different tissues is summarized in Table 3.

Further advances in understanding the roles of PHLDA1 in the mechanisms of apoptosis, autophagy, pyroptosis, and its stress-associated signals are essential for selectively manipulating these processes for therapeutic purposes.

## Involvement of PHLDA1 in differentiation

There have also been emerging associations of PHLDA1 with the differentiation process during neuronal development, adipogenesis, and tumorigenesis. Gomes et al. showed that the ectopic expression of PHLDA1 in hippocampal H19-7 cells was responsible for increased apoptosis under differentiating conditions, *i.e.*, fibroblast growth factor (FGF) present in the culture medium (Gomes et al. 1999). In another study, single-cell transcriptomic analysis revealed that *PHLDA1* was a highly expressed gene in precursors of retinal neurons (rods and cones) (Xiao et al. 2022). During differentiation of these photoreceptors, PHLDA1 expression was associated with increased expression of early photoreceptor markers and decreased expression of mature photoreceptor markers, as confirmed by immunostaining. Furthermore, *PHLDA1* silencing caused a decrease in early photoreceptor markers and a decrease in the number of photoreceptor precursors. The mechanism of photoreceptor development was explored, revealing that PHLDA1 is a negative regulator of AKT signalling. *PHLDA1* silencing increased the phosphorylation of AKT, and using AKT antagonist MK2206 increased photoreceptor differentiation. Noteworthy, adding insulin-like growth factor (IGF1) to the culture media not only upregulated the expression of PHLDA1 but also promoted photoreceptor differentiation, and this effect was reversed by PHLDA1 knockdown, indicating PHLDA1-dependent neuronal differentiation. In another study, microarray analysis showed that PHLDA1 expression is decreased during adipogenesis in 3T3-L1 cells (Burton et al. 2004). Also, Kim et al. showed that PHLDA1 is downregulated throughout the differentiation process of 3T3-L1 cells into mature adipocytes (Kim et al. 2021). Additionally, the induced expression of PHLDA1 inhibited the differentiation into adipocytes and the expression of adipogenic marker, peroxisome proliferator-activated receptor γ (PPARγ). Some important findings the group provided were that PHLDA1 directly interacted with PPARγ, as shown by co-immunoprecipitation and immunoblotting, and negatively regulated this protein. Furthermore, PHLDA1 expression was associated with the differentiation of germinative matrix cells in skin matrical tumors, as reported by Battistella et al. (Battistella et al. 2014a). Moreover, PHLDA1 downregulation was shown to affect differentiation-associated transcripts such as *ID1*, *ID2* (encoding inhibitors of differentiation 1, 2), *NTRK1*, *NTRK2* (encoding tropomyosin receptor A, B), and *NGF* (encoding nerve growth factor) in IMR-32 neuroblastoma cells (Durbas et al. 2016). Most importantly, the downregulation of PHLDA1 in IMR-32 increased the level of tropomyosin receptor kinase B (TRKB), a marker of poor prognosis in neuroblastoma (Brodeur et al. 2009; Durbas et al. 2016). It was also shown that *PHLDA1* silencing leads to differentiation-like phenotype in IMR-32 cells, manifested by a significant neurite outgrowth (Bugara et al. 2024). *PHLDA1* silencing decreases the level of secretogranin 2 (Bugara et al. 2024), a marker of neuronal differentiation into sympathetic neurons in neuroblastoma (Li et al. 2008). A more detailed analysis, including co-immunoprecipitation followed by mass spectrometry, indicated that PHLDA1 interacts with DDB1 and CUL4 associated factor 7/autism susceptibility gene 2 protein (DCAF7/AUTS2) complex in neuroblastoma (Bugara et al. 2024), essential for neuronal differentiation of mouse stem cells (Wang et al. 2018c). Further associations were found between the knockdown of PHLDA1 and the activation of the ErbB1 signalling pathway (Bugara et al. 2024), which is implicated in many processes, including differentiation (Wee and Wang 2017). Another study showed that PHLDA1 negatively controls cell differentiation in the MCF-7 breast cancer cells by inhibiting heregulin-dependent ErbB3 receptor activation (Magi et al. 2018). All these reports prove that PHLDA1 has an important role in neuronal, adipogenic, and tumorigenic differentiation.

Stem cells are undifferentiated or partially undifferentiated cells developing into various types of cells (Zakrzewski et al. 2019). Identifying PHLDA1 as a potential stem cell marker has attracted the attention of several groups. Ohyama et al. determined that PHLDA1 is highly expressed and localized in the hair follicle bulge, as indicated by microarray analysis and immunohistochemistry (Ohyama et al. 2006). PHLDA1 was also identified as a follicular stem cell marker in desmoplastic trichoepitheliomas, benign skin lesions originating from the hair follicle (Sellheyer and Krahl 2011). Immunohistochemical staining showed that nearly all desmoplastic trichoepitheliomas tested were PHLDA1-positive. Similarly, the increased PHLDA1 expression was reported by other groups in trichoblastoma, a tumor originating from germinative cells of the hair follicle (Battistella et al. 2014b; Misago et al. 2015). PHLDA1 was also marked to be a putative epithelial stem cell marker in the human small and large intestines (Sakthianandeswaren et al. 2011). It was overexpressed in normal colorectal tissues, HCT116, S480 colon cancer cell lines, and in all stages of colorectal cancer. Moreover, in IMR-32 neuroblastoma cells, *PHLDA1* silencing affected the expression of stem cell markers such as nestin and nanog (Bugara et al. 2024). Last, the single-cell RNA sequencing data revealed abundant PHLDA1 expression in cancer stem cells in Ewing sarcoma (Huang et al. 2022). Therefore, some undisputed evidence of the role of PHLDA1 as a stem cell marker exists.

## PHLDA1-miRNAs regulation in tumors

**Fig. 3:**
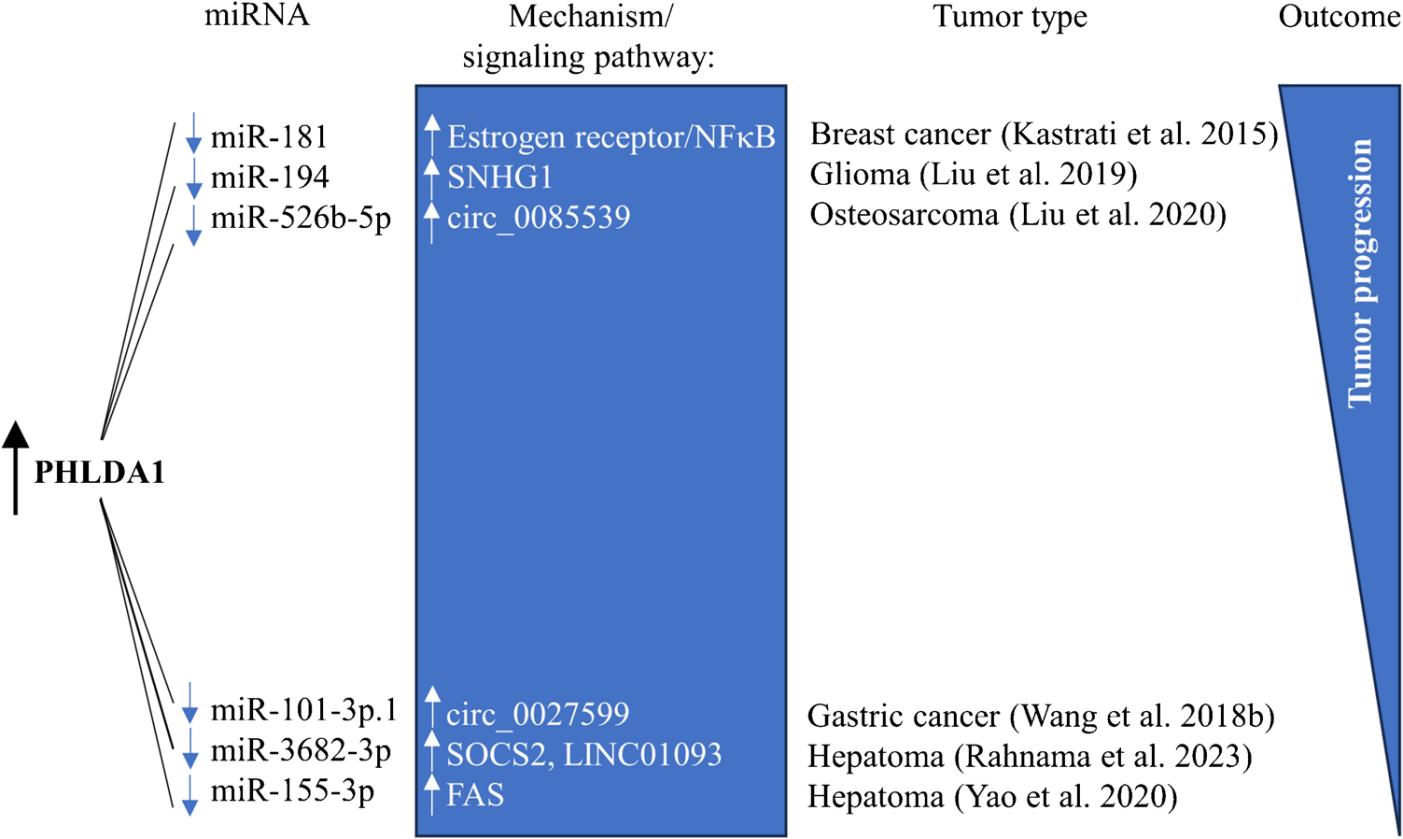
The role of PHLDA1-miRNAs regulation in the tumor progression. *PHLDA1* expression is negatively regulated with several miRNAs: miR-181, miR-194, miR-526b-5p, miR-101-3p.1, miR-3682-3p and miR-155-3p. *PHLDA1* mRNA-miRNAs interaction is modulated by long non-coding RNAs: SNHG1 – small nucleolar RNA host gene 1 and LINC01093 – long intergenic non-protein coding RNA 1093, and circular RNAs (circ_0085539, circ_0027599). SOCS2 – suppressor of cytokine signalling 2; FAS – TNF receptor superfamily member 6

Numerous recent publications have shown the importance of the interrelation between PHLDA1 and miRNAs in tumors. miRNAs are particularly interesting because of their involvement in carcinogenesis and cancer progression (Ling et al. 2011). There is ample evidence that miRNAs expression is dysregulated in human cancer by various mechanisms such as amplification or deletion of miRNA genes, abnormal transcriptional control of miRNAs, dysregulated miRNAs biogenesis machinery, and dysregulated epigenetic changes affecting miRNAs (Peng and Croce 2016). Some evidence about the regulation of PHLDA1 by miRNAs in various tumors exists in the literature and is presented as follows (Fig. 3). *PHLDA1* mRNA is a direct target of miR-181 in estrogen receptor-positive breast cancer cells (Kastrati et al. 2015). Firstly, it was reported that the downregulation of miR-181 by anti-miR inhibitors led to increased *PHLDA1* mRNA stability. Secondly, it was shown that PHLDA1 was increased in MCF-7 cells treated with 17β-estradiol acting via estrogen receptor and subsequently via NFκB pathway, whereas miR-181 was decreased upon this treatment. Thirdly, the group showed that PHLDA1 was increased in mammospheres of estrogen receptor-positive breast cancer. Its expression correlated with tumor metastasis (presented graphically as the increased tumor progression in Fig. 3). Therefore, it was concluded that PHLDA1 is upregulated directly by estrogen receptor/NFkB signalling and indirectly by inhibiting miR-181 and these events promote breast cancer progression. *PHLDA1* mRNA was also verified as a direct target of miR-194 in glioma (Liu et al. 2019). PHLDA1 is known to promote glioma progression, whereas miR-194 suppresses the proliferation and migration of glioma by regulating PHLDA1 (Liu et al. 2019; Wang et al. 2022a). Liu et al. demonstrated that small nucleolar RNA host gene 1 (SNHG1), which is a long non-coding RNA (lncRNA) involved in the development of multiple human tumors, including glioma, regulates *PHLDA1* expression by competitively binding to miR-194 (Liu et al. 2019). This type of regulation was well-reported in literature as the competition of lncRNAs for the binding of miRNAs to mRNAs of target genes, thus causing the release of mRNAs and consequently regulating the gene expression (Deng et al. 2019; Lan and Liu 2019; Wang et al. 2022b). The study above by Liu et al. confirmed this phenomenon, showing that SNHG1, being lncRNA, regulates *PHLDA1* expression by binding to miR-194 and acting as a molecular,,sponge”. These regulatory events were associated with glioma progression. Cicular RNAs (circRNAs) are non-coding RNAs and important regulators in cancer growth and progression (Bach et al. 2019). Similarly to lncRNAs, circRNAs were also reported to compete for miRNAs interaction, resulting in the occupation of their binding sites and, thus, the inhibition of miRNA functions (Memczak et al. 2013; Dragomir and Calin 2018). This mechanism was also observed in osteosarcoma and was again linked to PHLDA1 and miRNA regulation (Liu et al. 2020). The study identified *PHLDA1* mRNA as a target of miR-526b-5p in osteosarcoma cells. The inhibition of miR-526b-5p enhanced the level of PHLDA1 and was associated with osteosarcoma progression. The study showed that the miR-526b-5p-*PHLDA1* mRNA binding is regulated by circ_0085539, a circular RNA to be upregulated in this tumor. Furthermore, the negative regulation between circ_0085539 and miR-526b-5p was found in tested osteosarcoma tissues. As a matter of fact, circ_0085539 binds to miR-526b-5p, acting as the sponge of miRNA in osteosarcoma cells, indicating a mechanism similar to that reported for lncRNAs. Yet another group showed that *PHLDA1* mRNA is a target of miR-101 in gastric cancer, as shown by using bioinformatic analysis and luciferase reporter assay (Wang et al. 2018b). A similar mechanism based on sponging miR-101 by circ_0027599 to affect targeted genes in gastric cancer was also reported by this group. However, the consequences of circ_0027599/miR-101/PHLDA1 regulation on tumor development were drastically different because the observed regulation, contrary to the studies above, suppressed survival and metastasis in gastric cancer (presented graphically as the decreased tumor progression in Fig. 3). Thus, the decreased cell proliferation and migration ability was associated with PHLDA1 overexpression and upregulation of circ_0027599. Yet another example of sponging miRNA, and the last described here in relation to PHLDA1, was found in hepatoma (Rahnama et al. 2023). Bioinformatic analysis predicted that long intergenic non-protein coding RNA 1093 (LINC01093) binds competitively to miR-155-3p and alters PHLDA1 expression after milk thistle nano-micelle formulation treatment. This herbal drug decreased the expression of miR-155-3p and caused the increase of PHLDA1 expression together with suppressor of cytokine signalling 2 protein (SOCS2). This LINC01093/miR-155-3p/PHLDA1 regulatory axis led to decreased cell proliferation and induced apoptosis of hepatoma cells, therefore adding to the group of results demonstrating decreased potential of tumor progression. The second study on hepatoma revealed that *PHLDA1* mRNA is also a direct target of miR-3682-3p (Yao et al. 2020). The decreased expression of miR-3682-3p was associated with increased PHLDA1 levels in hepatoma cells, pointing once again to the negative correlation between PHLDA1 and miRNAs. This was also accompanied by the increased expression of TNF receptor superfamily member 6 (FAS). Additionally, knocking down miR-3682-3p enhanced apoptosis and decreased tumor growth in nude mice. The evidence presented above shows the negative regulation between PHLDA1 and several miRNAs, with the evident involvement of lncRNAs and circRNAs affecting this *PHLDA1* mRNA/miRNAs interaction. The consequences of this regulation could be dual, either promoting tumor progression or suppressing it, similar to the role of PHLDA1 described in tumors.

## PHLDA1 is linked to inflammation and neuroinflammation

PHLDA1 has been demonstrated to play an important role in inflammatory response. During infection, toll-like receptors (TLRs) respond to the variety of lipopeptides (LPs) or lipopolysaccharides (LPS) present in tissues and the bloodstream and trigger proinflammatory reactions facilitating eradication of the invading bacteria (Ciesielska et al. 2021). PHLDA1 was shown to be up-regulated in LPS-stimulated RAW264.7 cells (Jiao et al. 2016). *PHLDA1* silencing increased the proliferation and cell cycle progression of RAW264.7 cells treated with LPS. Several groups further explored the mechanism, which showed that PHLDA1 upregulation is involved in lipopeptides/LPS response through TLR signaling in macrophages (Lyu et al. 2016; Park et al. 2023). Microarray analysis performed by Lyu et al. showed that *PHLDA1* gene is involved in the TLR2 pathway in RAW264.7 and bone marrow-derived macrophages (Lyu et al. 2016). PHLDA1 expression was also induced by synthetic lipopeptide (Pam_3_CSK_4_), which is a TLR2 ligand that mimics the amino terminus of LPS. The group established that JAK2/ERK1/2/STAT3 pathway participates in Pam_3_CSK_4_-stimulated up-regulation of PHLDA1 by performing a series of experiments using specific JAK2 and ERK1/2 inhibitors (AG490 and U0126, respectively) and the knockdown of STAT3. PHLDA1 induction by LPS stimulation was also shown to be mediated by the TLR2/4 signaling pathway followed by the activation of its downstream effectors, such as myeloid differentiation primary response protein (MyD88) and TNF receptor-associated factor 6 (TRAF6) in bone marrow-derived macrophages (Park et al. 2023). LPS-stimulated PHLDA1 expression was inhibited after treatment with JNK, ERK, P38, and NFκB inhibitors (JNK-IN-8, PD98059, SB203580, and Bay11-7085, respectively), indicating the involvement of MAPKs and NFκB pathways in the observed response. Additionally, PHLDA1 was shown to interact with forkhead box O (FoxO1) transcription factor, as shown by immunoprecipitation and immunoblotting. Furthermore, PHLDA1^+/+^ bone marrow-derived macrophages were characterized by increased levels of proinflammatory cytokines such as IL-1β, IL-6 and TNFα, compared to PHLDA1-deficient ones, as assessed by cytokine expression array and ELISA. On the contrary, Peng et al. found that PHLDA1 repressed LPS-induced proinflammatory cytokine production in RAW264.7 (Peng et al. 2022). IL-1β, IL-6 and TNFα were decreased after overexpression of PHLDA1 in both bone marrow derived macrophages and RAW264.7 cells, while the *PHLDA1* silencing reversed the effect. The group found a negative regulation between PHLDA1 expression and TLR4/MyD88/MAPK signaling pathway in RAW264.7 cells, contradicting the results obtained by Lyu et al. and Park et al. Additionally, the overexpression of PHLDA1 obtained by Peng et al. decreased the activity and nuclear translocation of NFκB and AP-1 transcription factors as assessed by immunofluorescence. Therefore, the regulatory mechanism remains unclear, and the opposite regulation needs a further explanation. Interestingly, PHLDA1 was shown to bind to a toll-interacting protein (Tollip), a ubiquitin-binding protein that interacts with several toll-like receptors and their downstream targets (Peng et al. 2022). The group showed that the interaction between PHLDA1 and Tollip suppressed LPS-stimulated TLR4 signaling. The association between Tollip and PHLDA1 was also made by our group in IMR-32 neuroblastoma cells with PHLDA1 downregulation, as indicated by mass spectrometry (Bugara et al. 2024). The expression of Tollip was explicitly found in *PHLDA1*-silenced IMR-32 cells. Therefore, the influence of PHLDA1 and Tollip regulation is also worth further investigation in the context of cancer.

There are also reports on the role of PHLDA1 in several inflammatory diseases, such as kidney and lung injury, lung contusion, and intestinal colitis. PHLDA1 expression was positively correlated with inflammation and the production of proinflammatory cytokines in mice models of acute kidney and lung injury, as reported by two studies (Gong et al. 2022; Meng et al. 2023). The former study associated kidney inflammation with activating the JNK/ERK pathway, whereas *PHLDA1* knockdown led to the deactivation of JNK and ERK kinases, as demonstrated by decreased phosphorylation of JNK and ERK. The latter study correlated increased expression of PHLDA1 with increased pyroptosis in acute lung injury, as shown by inducing key pyroptosis markers such as NLRP3, ASC, active forms of caspase 1, and gasdermin D. Similar observation was made by Wang et al., who showed that increased PHLDA1 levels in mice model correlated with increased lung injury (contusion). Indeed, PHLDA1 enhanced proinflammatory cytokine response, and increased levels of TLR2 (Wang et al. 2016). PHLDA1 was also found to be a positive regulator of intestinal inflammation and pyroptosis, and the knockdown of PHLDA1 in mice model of colitis, was characterized by the decreased proinflammatory response (Yang et al. 2023). Additionally, the knockdown of PHLDA1 evoked similar events in mice after dextran sulfate sodium-induced colitis (Jeon et al. 2022).

Neuroinflammation is an inflammatory response centralized within the brain and spinal cord (DiSabato et al. 2016). The role of PHLDA1 in neuroinflammation was found in C57BL/6 mice model after subarachnoid hemorrhage, bleeding in the space between the brain and the membrane that covers it (Lai et al. 2023). Immunofluorescence showed that PHLDA1 expression in microglia was increased after this emergency. The study showed that PHLDA1 deficiency reduced proinflammatory cytokines production, NLRP3 inflammasome signaling, and neuronal apoptosis after subarachnoid hemorrhage. PHLDA1 knockdown in BV2 murine microglia cells also resulted in reduced production of proinflammatory cytokines following LPS stimulation (Han et al. 2020). Additionally, it decreased TLR/MyD88 signaling and, subsequently, JNK/ERK and NFκB pathways. However, in contrast to the previous report, PHLDA1 knockdown did not affect apoptosis of BV2 cells. Overall, PHLDA1 role is also relevant in neuroinflammation.

## Ischemia/reperfusion injury and the involvement of PHLDA1

Ischemia/reperfusion injury (IRI) is a common feature of ischemic stroke, which occurs when blood supply is restored after a period of ischemia (Lin and Wang 2016). The role of PHLDA1 in this tissue damage was frequently reported. The PHLDA1 facilitated neuronal damage in a brain ischemia/reperfusion injury after oxygen–glucose deprivation/reoxygenation, OGD/R (Sun et al. 2023). PHLDA1 overexpression caused the increased apoptosis of hippocampal neurons obtained from rat brains, as detected via the TUNEL method. Next, it was shown that the observed apoptosis is inhibited by helix-loop-helix family member e40 (BHLHE40), which represses *PHLDA1* gene transcription by binding to its promotor. It was also accompanied by decreased level of BAX and increased level of BCL-2, key features of apoptosis inhibition. Another report by Liu et al. also confirmed that PHLDA1 increased neuronal apoptosis during ischemia/reperfusion injury induced by OGD/R (Liu et al. 2023). The group further explored the mechanism of this regulation, showing that knockdown of PHLDA1 inhibited apoptosis. These OGD/R-induced events were associated with increased nuclear translocation of peroxisome proliferator-activated receptor γ (PPARγ). Further confirmation of PPARγ involvement was achieved when the results showed that treating neurons with PPARγ antagonist, GW9662, induced apoptosis. The same results were also obtained independently after PHLDA1 overexpression. These findings were further expanded by showing that the increased levels of PHLDA1 in ischemia/reperfusion injury are responsible for inducing apoptosis via enhancing ER stress and mitochondrial dysfunction, events that were reported by numerous groups so far in cancer and related to PHLDA1 action. A similar involvement of PHLDA1 in inducing neuronal apoptosis was also confirmed by Yang and Chen (Yang and Chen 2021). They indicated the mechanism based on supressing glycogen synthase kinase 3β/nuclear factor erythroid 2-related factor 2 (GSK-3β/NRF2) pathway that plays a vital role in neuroprotection (Cuadrado et al. 2018; Sotolongo et al. 2020; Yang and Chen 2021; Soni and Kumar 2022).

Furthermore, the involvement of PHLDA1 in ischemia/reperfusion injury was reported in human and rat cardiomyocytes. Oxidative stress is essential in initiating IRI (De Vries et al. 2013). The group of Guo et al. showed that overexpression of PHLDA1 caused the enhanced apoptosis of rat H9c2 cardiomyocytes exposed to oxidative stress-inducing H_2_O_2_, as evidenced by increased levels of cleaved caspase 3 and PARP (Guo et al. 2020). The decreased mitochondrial membrane potential and increased production of reactive oxygen species were also noted after H_2_O_2_ treatment. The study indicated that PHLDA1 binds to BAX and enhances cardiomyocyte damage by inhibiting BAX degradation in proteasome. The function of PHLDA1 was further explored in tissues isolated from the hearts of rats subjected to ischemia/reperfusion (IRI-treated group) or perfusion alone (control group). Undamaged myocardial tissue and decreased apoptosis were observed after PHLDA1 knockdown, indicating that PHLDA1 is involved in IRI at the tissue level. Furthermore, the expression of PHLDA1 was also elevated in both human ischemic cardiac cell lines and the ischemic rat model (Wang et al. 2018a). Overexpression of PHLDA1 promoted apoptosis of cardiac muscle cells and was associated with inducing BAX and P53. The negative correlation between PHLDA1 and AKT was also described, confirming the opposite regulation of these two proteins observed in tumors. Interestingly, the previously described regulation between PHLDA1 and miR-194 was also responsible for hepatic ischemia/reperfusion injury (Luo et al. 2021). The role of PHLDA1 in promoting ischemia/reperfusion injury, as revealed by the aforementioned studies, is evident. PHLDA1 exerts its effect in IRI by activating apoptosis-related signalling pathways. Accumulated evidence emphasizes the potential of downregulating PHLDA1 in therapy of ischemia/reperfusion injury.

**Fig. 4:**
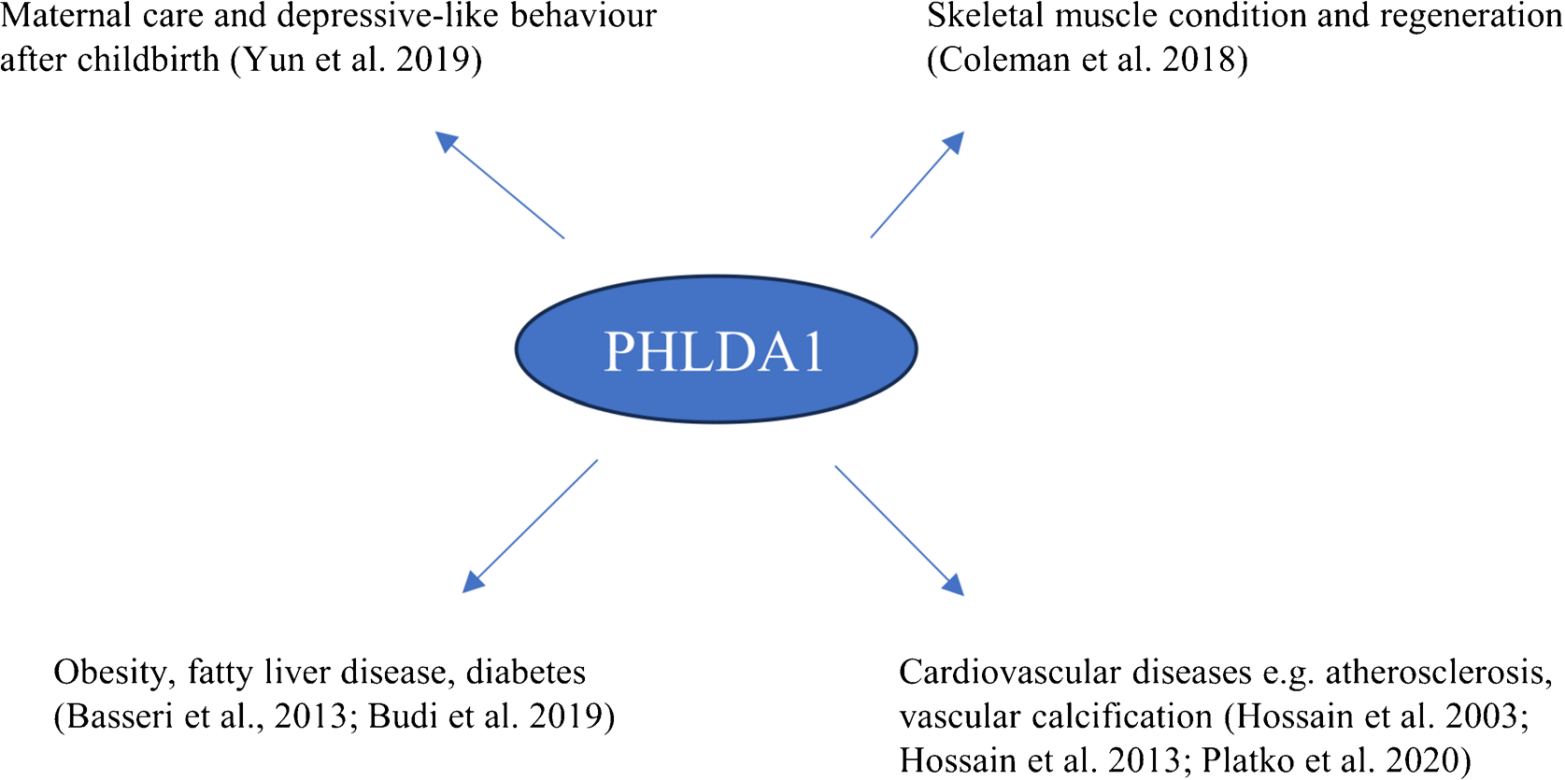
The involvement of PHLDA1 in other processes and diseases

The roles of PHLDA1 in other processes and diseases were also described in the literature (Hossain et al. 2003, 2013; Basseri et al. 2013; Coleman et al. 2018; Budi et al. 2019; Yun et al. 2019; Platko et al. 2020) and are presented collectively in Fig. 4. However, they are beyond the scope of this review.

## Conclusions

The diverse roles of PHLDA1 are still reported and are under ongoing investigations. New functions of PHLDA1 surfaced throughout the last three decades, indicating that PHLDA1 is a multifunctional protein. PHLDA1 is shown to exert pleiotropic effects that depend on the cellular context. The level of PHLDA1 expression and stimulation manner determines tissue-specific activities. The *PHLDA1* gene and PHLDA1 protein are regulated by various treatments and stimuli, including cell differentiation-, mitogenic-, and ER stress agents, with several implications related to cell fate. PHLDA1 upregulation or downregulation affects biological processes such as apoptosis, proliferation, autophagy, ER stress, pyroptosis, or differentiation. Because the findings about the role of PHLDA1 in autophagy are sparse, further studies should be undertaken to explore how PHLDA1 regulates this process. New insights into the role of PHLDA1 in mitochondria regulation in the context of mitophagy (mitochondria degradation) are worthy of further investigation. The involvement of PHLDA1 in mitophagy mediated by FUN14 Domain-Containing Protein 1 (FUNDC1) in ischemic brain injury has recently been reported (Jiang et al. 2024). Furthermore, the role of the regulation between PHLDA1 and Tollip remains to be elucidated, as both proteins are reported to be involved not only in antimicrobial response but also in the process of mitophagy, as the findings by some groups suggest (Peng et al. 2022; Shin and Chung 2022; Bugara et al. 2024; Jiang et al. 2024). PHLDA1 functions were implicated in a vast number of pathophysiological conditions such as cancer, ischemia/reperfusion injury, and lipid disorders. The dichotomous role of PHLDA1 in cancer is evidenced more widely, being both pro- and antiapoptotic depending on the tumor type, cell milieu, and the insult. New experimental studies are required to establish the *PHLDA1* role as a tumor suppressor gene or oncogene and its impact on tumor progression. Frequently reported findings regarding PHLDA1 regulation via RAS/MAPK and AKT signalling pathways consolidate the role of PHLDA1 in tumors. Further in vivo studies are warranted using animal models that will clarify the PHLDA1 role in normal and cancer cells. Finally, the significance of PHLDA1 as a prognostic factor and a potential therapeutic target needs to be further explored.

## Data Availability

No datasets were generated or analysed during the current study.

## Abbreviations

PHLDA1: Pleckstrin homology-like domain, family A, member 1
MAPKs: Mitogen-activated protein kinases
ERK1/2: Extracellular signal-regulated kinase 1/2
PARP: Poly (ADP-ribose) polymerase
TRKB: Tropomyosin receptor kinase B
OS: Overall survival
ER: Endoplasmic reticulum
NLRs: Nod-like receptors
NLRP3: NLR family pyrin domain-containing 3
ASC: Apoptosis-associated speck-like proteins containing a CARD
PPARγ: Peroxisome proliferator-activated receptor γ
IRI: Ischemia/reperfusion injury
